# Functional defects in CD4^+^ CD25^high^ FoxP3^+^ regulatory cells in ankylosing spondylitis

**DOI:** 10.1038/srep37559

**Published:** 2016-11-30

**Authors:** Huifang Guo, Ming Zheng, Kui Zhang, Fengfan Yang, Xin Zhang, Qing Han, Zhi-Nan Chen, Ping Zhu

**Affiliations:** 1 Department of Clinical Immunology, Xijing Hospital, The Fourth Military Medical University, No. 127 West Changle Road, Xi’an, Shaanxi Province, People’s Republic of China; 2 Department of Cell Biology, Fourth Military Medical University, China.; 3 National Translational Science Center for Molecular Medicine, Xi’an 710032, China

## Abstract

Forkhead box P3 (FoxP3)-positive regulatory T cells (Tregs) play a pivotal role in the preservation of self-tolerance, and Treg dysfunction has been implicated in many autoimmune diseases. Whether and how Tregs participate in the pathogenesis of ankylosing spondylitis (AS) has not been fully elucidated. Here, we investigated Treg function and found that Tregs in peripheral blood (PB) from patients with active AS had lower FoxP3 mean fluorescence intensity (MFI) than those from healthy controls and could not fully suppress naïve T cell (Tn) proliferation. We also studied the mechanisms underlying PB Treg dysfunction in this context and found that PB Tregs failed to effectively utilize IL-2 and had relatively little STAT5 phosphorylation in active AS. Moreover, PB Tregs from patients with active AS exhibited greater CpG island methylation in the CNS2 region of the *FOXP3* gene. Therefore, our findings indicate that functional defects in Tregs are present in AS. Abnormal IL-2 signalling and aberrant CNS2 epigenetic control induced functional defects in PB Tregs and represents a potential new mechanism for AS pathogenesis. These findings may aid the design of new treatment approaches for AS.

Ankylosing spondylitis (AS) is a chronic autoimmune inflammatory disease. Traditionally, AS was thought to be associated with human leukocyte antigen B27 (HLA-B27)^1^,^2^; however, more recent research has demonstrated that AS is also a T lymphocyte-associated disease and that CD4^+^ T cells and their subsets may participate in the development of AS^3^,^4^,^5^,^6^. Most studies have suggested that Forkhead box P3 (FoxP3)-positive regulatory T cells (Tregs) play a role in the aetiology of AS^5^,^7^,^8^. However, whether and how peripheral blood (PB) Tregs control AS severity are questions that remain unresolved.

Both the number and function of PB Tregs are crucial for the suppression of inflammatory and autoimmune pathology, and disruptions in both factors have been implicated in the pathogenesis of many inflammatory and autoimmune diseases^9^, including type 1 diabetes (T1D)^10^ and multiple sclerosis (MS)^11^. However, studies of AS phenotypes have produced controversial results. Some reports have shown that the percentage of PB Tregs does not change in AS^5^,^7^,^8^, whereas others have shown the opposite effect^12^,^13^. However, some function-related phenotypes, such as FoxP3 mean fluorescence intensity (MFI), have never been evaluated. Additionally, few studies have investigated the suppressive function of PB Tregs in AS. Given the importance of PB Treg function in autoimmune disorders, further investigations into the role of PB Tregs in AS are warranted.

Treg functions, especially immunosuppressive functions, are mainly regulated by the expression of the transcription factor FoxP3^14^. Two critical mechanisms have been proposed to explain how stable FoxP3 expression is maintained in Treg; these include interleukin-2 (IL-2) signalling and CNS2 methylation^15^. Alterations in IL-2 signalling decrease FoxP3 expression, which is further associated with impaired Treg proliferation in subjects with relapsing-remitting multiple sclerosis (RRMS)^9^. However, it remains unknown whether changes in IL-2 signalling in PB Tregs drive AS pathogenesis. Additionally, no studies have investigated the roles of CNS2 methylation and PB Treg function in autoimmune disorders such as AS. Therefore, how these factors affect patients with AS warrants further investigation.

To investigate the issues described above, the present study was designed to measure the frequencies and examine the functions of various PB CD4^+^ T cell subsets, especially the suppressive function of PB Tregs, in AS and to elucidate the mechanisms that drive PB Treg function, such as IL-2 signalling and CNS2 methylation. Elucidation of the mechanisms through which Tregs participate in the development of AS will increase understanding of AS, a T cell-associated disease, and lead to better preventative measures.

## Results

### Proliferation, apoptosis and Th17 cell differentiation of naïve PB T cells (Tns) were similar between patients with active AS and healthy controls

**Figure 1:**
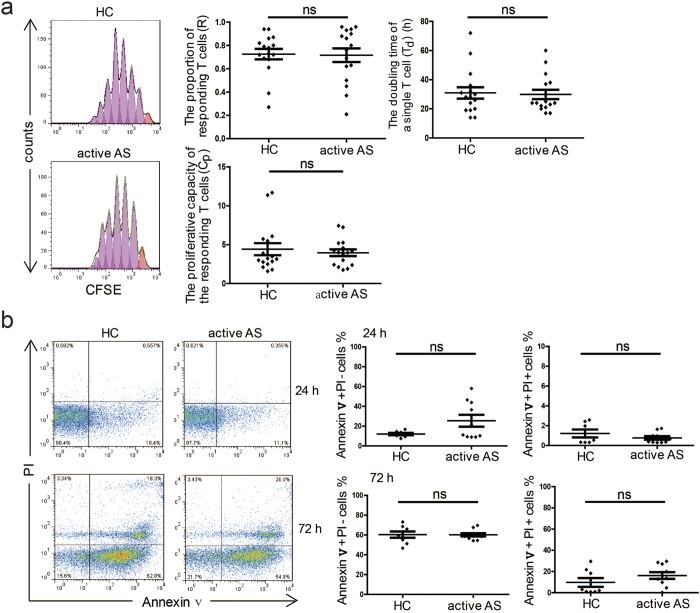
Proliferation and apoptosis of naïve T cells. (**a**) Naïve T cells (Tns) from healthy controls (HCs) and patients with active ankylosing spondylitis (AS) (each n = 16) were labelled with 5, 6-carboxyfluorescein succinimidyl ester (CFSE) and cultured in the presence of anti-CD3/CD28 beads (Tns:beads = 1:1). On day 5, the cells were assessed, and the R, T_d_ and C_p_ values were calculated. The histograms show the CFSE dilution in representative samples (left). (**b**) Tns from HCs (n = 8) and patients with active AS (n = 10) were cultured in the presence of anti-CD3/CD28 beads (Tns:beads = 1:1). The percentages of Annexin V^+^ PI^−^ and Annexin V^+^ PI^+^ cells within the Tn populations were assessed by flow cytometry after 24 or 72 hours of culture. The representative dot plots show the percentages of Annexin V^+^ PI^−^ and Annexin V^+^ PI^+^ cells in the Tn populations (left). R: the proportion of the precursor sample pool that, responded to stimulation by dividing. T_d_: the time required for the average responding T cells to achieve a single cell division. C_p_: the proliferative capacity of the responding T cells. p < 0.05 indicates significance (unpaired Student’s *t*-test); ns: not significant.

The proliferative capacity of naïve PB Tns in active AS was determined 5 days following stimulation with anti-CD3/CD28 beads. The results are expressed as R, T_d_ and C_p_ values, where R represents the proportion of the precursor sample pool that responded to stimulation by dividing; T_d_ prepresents the time required for the average responding T cell to achieve a single cell division, i.e., the doubling time; and C_p_ represents the proliferative capacity of the responding T cells for each sample^16^,^17^. We found no significant differences in any of these values between PB samples collected from patients with active AS and those collected from healthy controls (each n = 16) (Fig. 1a). There were also no differences in the percentages of Annexin V^+^ PI^−^ cells and Annexin V^+^ PI^+^ cells between the PB samples collected from patients with active AS (n = 10) and those collected from healthy controls (n = 8) either 24 hours or 72 hours after Tn stimulation (Fig. 1b). In addition, IL-23 R^+^ cell frequency within PB CD4^+^ T cell populations and CD4^+^ IL-17a^+^ Th17 cell frequency after induction exhibited no difference between patients with active AS and healthy controls (see Supplementary Fig. S1). Taken together, these data indicate that no differences were found in the proliferation, apoptosis or Th17 cell differentiation of PB Tns between patients with active AS and healthy controls.

### Classical Tregs from patients with active AS exhibit reduced FoxP3 MFI

**Figure 2:**
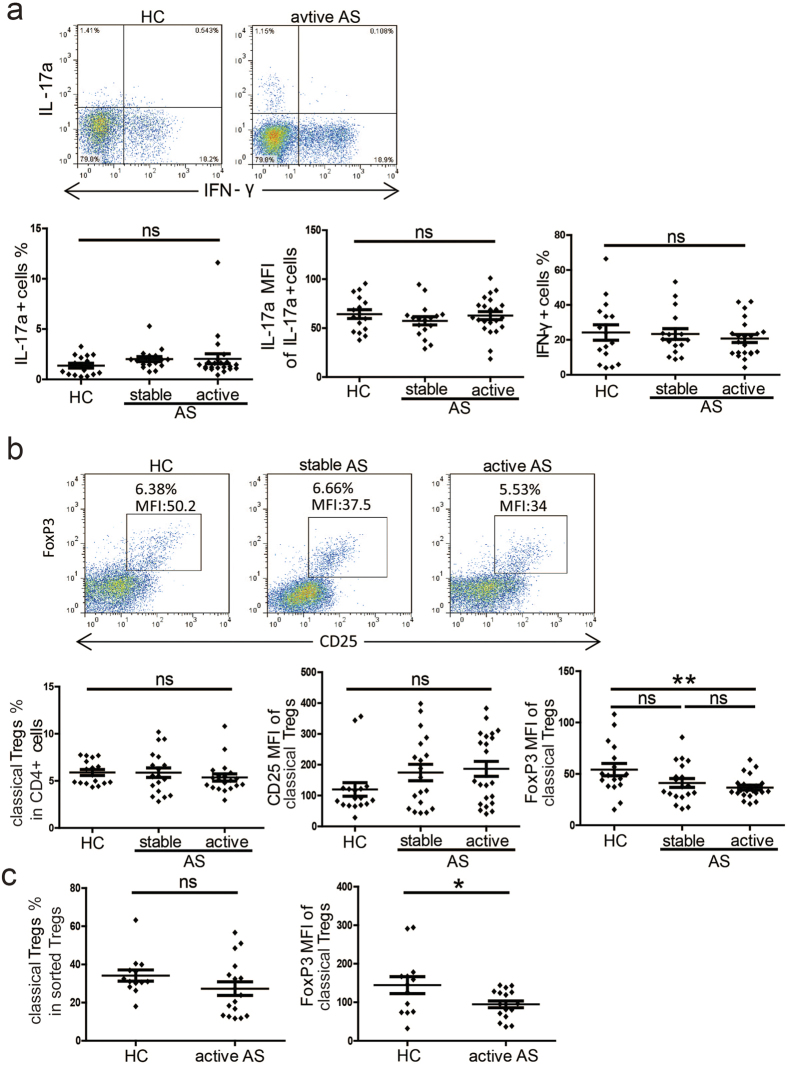
Percentages of Th1/Th17 cells and Tregs. (**a**) Peripheral blood mononuclear cells (PBMCs) from HCs (n = 16), patients with stable AS (n = 17) and patients with active AS (n = 22) were stimulated with phorbol myristate acetate (PMA) and ionomycin for 5 hours in the presence of GolgiStop, and the percentages of Th1/Th17 cells within the CD4^+^ CD45RO^+^ memory T cell populations as well as the IL-17a mean fluorescence intensity (MFI) of the CD4^+^ IL^-^17a^+^ cells were determined. Representative dot plots are shown (upper panel). (**b**) The percentages of classical Tregs within the CD4^+^ T cell populations in PBMCs and the CD25 MFI and FoxP3 MFI of classical Tregs were determined in HCs (n = 17), patients with stable AS (n = 19) and patients with active AS (n = 20). Representative dot plots are shown (upper panel). (**c**) The percentages of classical Tregs within the sorted Tregs and the FoxP3 MFI of the classical Tregs were determined in HCs (n = 24) and patients with active AS (n = 21). p < 0.05 indicates significance (unpaired Student’s *t*-test); *p < 0.05; **p < 0.01; ns: not significant.

We next measured the percentages of Th1/Th17 cells within CD4^+^ CD45RO^+^ memory T cell populations in PB samples collected from patients with AS and healthy controls. We found no significant differences in the percentages of Th1/Th17 cells among PB samples from patients with active AS (n = 22), patients with stable AS (n = 17) and healthy controls (n = 16). Additionally, there were no significant differences in Th17 cells IL−17a MFI values (Fig. 2a).

Furthermore, we found no significant differences in the percentages of classical Tregs within PB CD4^+^ T cell populations among patients with active AS (n = 20), patients with stable AS (n = 19) and healthy controls (n = 17). Additionally, no differences were found in CD25 MFI in classical Tregs. However, we observed a significant decrease in FoxP3 MFI in the PB classical Tregs in patients with active AS compared with healthy controls (p = 0.0054) (Fig. 2b). Moreover, we found no significant difference in the percentages of classical Tregs within sorted Treg populations between patients with active AS (n = 21) and healthy controls (n = 24), but we did observed a significant decrease in FoxP3 MFI in classical Tregs from patients with active AS (p = 0.0301) (Fig. 2c).

Taken together, these data demonstrate that AS does not affect percentages of Th1/Th17 cells. However, the lower FoxP3 MFI values measured in the PB classical Tregs suggests that these cells do not function normally in active AS^18^.

### Defects in the suppressive function of Tregs in patients with active AS

**Figure 3:**
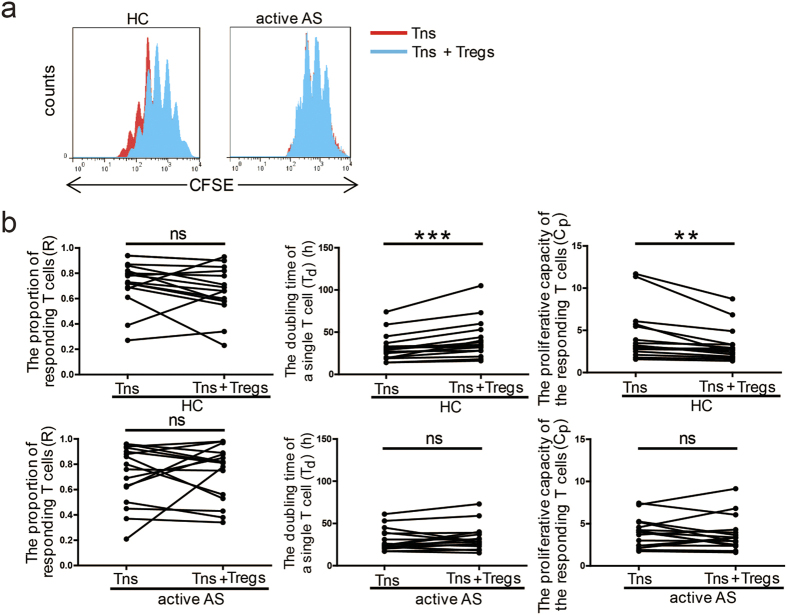
Suppression of Tn proliferation by Tregs. Histograms show the CFSE dilution in representative samples from a HC and a patient with active AS, and the proliferation curve of Tns cultured alone (red) was overlaid with the curve for Tns co-cultured with Tregs (blue) (**a**). Tns from HCs and patients with active AS (each n = 16) were labelled with CFSE and co-cultured with sorted Tregs (Tns:sorted Tregs = 2:1) in the presence of anti-CD3/CD28 beads (Tns:beads = 1:1). On day 5, Tn proliferation was assessed by flow cytometry, and the R, T_d_ and C_p_ values for the Tns were calculated (**b**). p < 0.05 indicates significance (paired Student’s *t*-test); *p < 0.05; **p < 0.01; ***p < 0.001; ns: not significant.

We next investigated the suppressive function of PB Tregs sorted from patients with active AS by measuring the ability of the sorted cells to suppress Tn proliferation *in vitro*. After Tregs and Tns were sorted from the PB of patients with active AS and healthy controls (each n = 16), the Tns were labelled with CFSE and co-cultured with the sorted Tregs (Tns:Tregs = 2:1) in the presence of anti-CD3/CD8 beads (Tns:beads = 1:1). Tn proliferation was assessed on day 5 by gating on CFSE^+^ cells (Fig. 3a). The presence of sorted PB Tregs from healthy controls led to significantly prolonged T_d_ (p = 0.0006) and significantly decreased C_p_ (p = 0.0035), although the R value did not change (Fig. 3b). These results confirm that PB Tregs exhibit suppressive functions toward a variety of immune cells, including Tns^19^. However, the presence of PB Tregs sorted from patients with active AS did not effectively inhibit Tn proliferation and led to no changes in R, T_d_ (p = 0.3319) or C_p_ (p = 0.5757) values (Fig. 3b). Taken together, these data suggest that PB Tregs in patients with active AS are defective in their suppressive function.

It has previously been shown that CD39 expression on pre-existing memory T cells confers protection against ATP-induced apoptosis and reflects highly active cells^20^. We found that patients with active AS (n = 9) had a higher percentage of CD39^+^ cells within their Tn populations than healthy controls (n = 14) based on co-culture of Tns with Tregs (see Supplementary Fig. S2). These results indicate that patients with active AS have more active Tns than healthy controls after co-culture with Tregs. This higher Tn activity may be caused by the weak suppressive function of PB Tregs in active AS.

### Patients with active AS showed greater IL-2 accumulation in the supernatant of co-cultured Tregs and Tns than healthy controls

**Figure 4:**
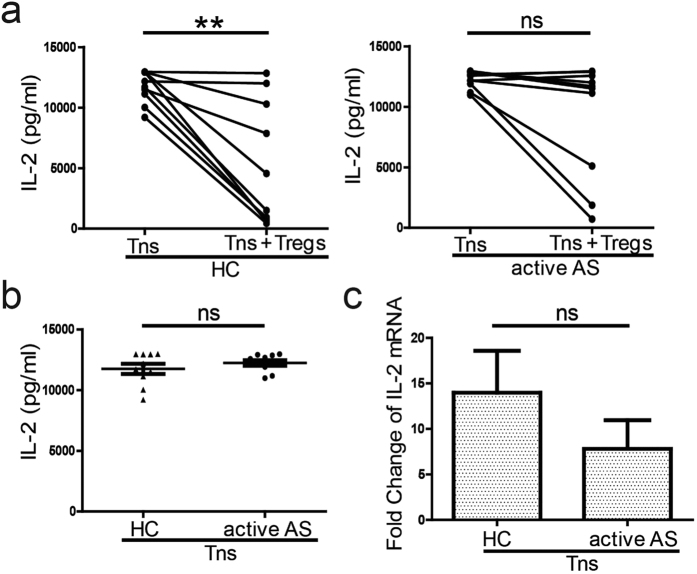
Concentrations of interleukin-2 (IL-2) in cell co-culture supernatants and *IL-2* gene expression in Tns. (**a**) and (**b**) Tns from HCs and patients with active AS (each n = 10) were treated and cultured as described in Fig. 3. On day 5, supernatants were collected. The IL-2 levels in the supernatant of Tns cultured alone or co-cultured with sorted Tregs were assessed (**a**). The IL-2 levels in the supernatants of the Tns cultured alone were assessed (**b**). (**c**) Tns from HCs (n = 6) and patients with active AS (n = 6) were treated and cultured as described in Fig. 3 On day 5, the cells were collected and RNA was extracted and reverse-transcribed into cDNA. IL-2 mRNA expression was assessed via real-time quantitative polymerase chain reaction (qPCR). Unpaired Student’s *t*-test was used for comparisons between HCs and patients with active AS; otherwise, paired Student’s *t*-test was utilized. p < 0.05 indicates significance; **p < 0.01; ns: not significant.

The above results suggest that patients with active AS harbour PB Tregs with defective suppressive ability. Several studies have demonstrated the importance of IL-2 for Treg function^21^. Therefore, we measured IL-2 levels in the co-culture supernatants of PB Tregs and Tns from patients with active AS and healthy controls (each n = 10). The presence of sorted PB Tregs from healthy controls led to a significant reduction in IL-2 levels (p = 0.0013). However, the presence of sorted PB Tregs from patients with active AS did not change the IL-2 level in the co-culture supernatant (p = 0.0510) (Fig. 4a). Additionally, no differences were found in supernatant IL-2 levels between healthy controls and patients with active AS when Tns were cultured alone (Fig. 4b). These results suggest that IL-2 is more abundantly produced in Tn co-culture supernatants form patients with active AS.

We also evaluated *IL-2* gene expression in PB Tns from patients with active AS and healthy controls (each n = 6) after co-culture with sorted PB Tregs, as described for the functional suppression assays above. We found no difference in the fold changes in *IL-2* gene expression between PB Tns from healthy controls and those from patients with active AS (Fig. 4c). These results suggest that IL-2 levels in cell co-culture supernatants from patients with active AS are a product of reduced IL-2 usage by PB Tregs from these patients.

Additionally, we assessed the concentrations of TGF-β, granzyme B and IL-10 in the co-culture supernatants on day 5. No differences were found in these concentrations between patients with active AS and healthy controls (each n = 10) (see Supplementary Fig. S3). These results are consistent with our *IL-10* and *TGF-β* expression results from isolated Tregs (see Supplementary Fig. S4) and reveal that there are no differences in cytokine expression between sorted PB Tregs from healthy controls and those from patients with active AS.

### Reduced STAT5 phosphorylation in sorted PB Tregs from patients with active AS

We next investigated STAT5 expression in sorted PB Tregs isolated from patients with active AS and from healthy controls. After sorting, PB Tregs were stimulated with IL-2. The percentage of STAT5^+^ (pho-STAT5^+^) cells in the sorted Treg populations increased rapidly within 5 minutes of stimulation. The percentage then gradually declined, reaching a plateau after 30 minutes (see Supplementary Fig. S5). Based on these results, 0, 5 and 30 minutes were the best time points for detection.

**Figure 5:**
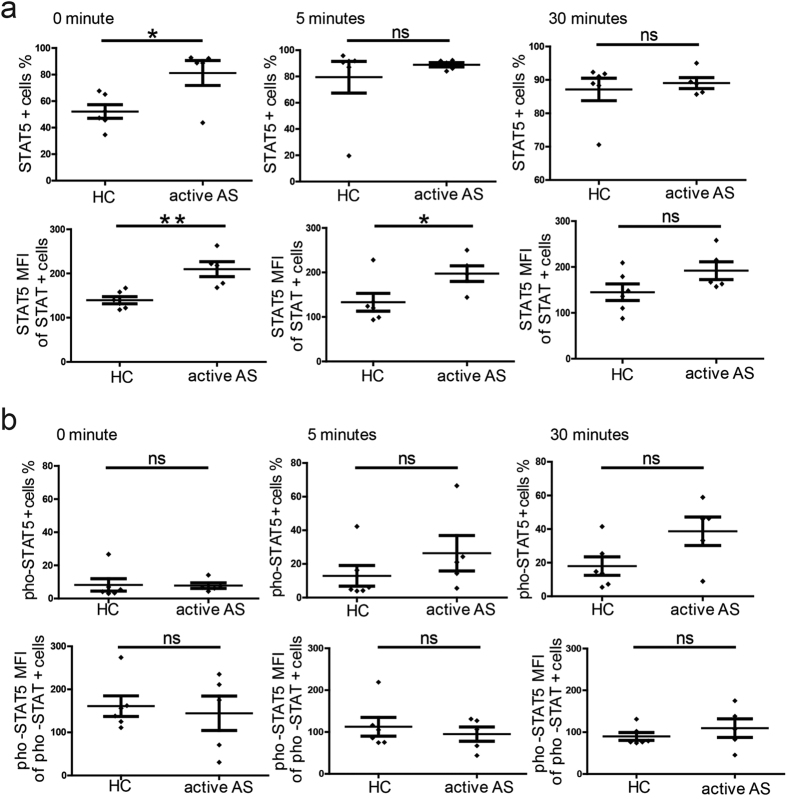
Phosphorylation levels of signal transducer and activator of transcription 5 (STAT5) in Tregs following stimulation with interleukin-2 (IL-2). Sorted Tregs from HCs (n = 6) and patients with active AS (n = 5) were starved in serum-free medium for 2 hours and stimulated with IL-2 for different periods of time (0, 5 and 30 minutes). The percentages of STAT5^+^ T cells and the STAT5 MFI of the STAT5^+^ Tregs were assessed (**a**) in addition to the percentages of phosphorylated-STAT5 (pho-STAT5)^+^ T cells and the pho-STAT5 MFI of the pho-STAT5^+^ Tregs (**b**). p < 0.05 indicates significance (paired Student’s *t*-test); *p < 0.05; **p < 0.01; ns: not significant.

We next investigated STAT5 expression in PB Tregs sorted from patients with active AS (n = 5) and healthy controls (n = 6) after stimulation with IL-2 for 0, 5 and 30 minutes. We found that patients with active AS had a higher percentage of STAT5^+^ cells than healthy controls at the time point of 0 minutes. However, no differences were found at 5 or 30 minutes. Additionally, the sorted PB Tregs from patients with active AS exhibited higher STAT5 MFI values compared to those from healthy controls at 0 and 5 minutes following IL-2 stimulation. No differences were found at the 30-minute time point (Fig. 5a).

Because STAT5 function is reflected in the protein’s phosphorylation status, we next investigated pho-STAT5 protein expression in sorted PB Tregs from patients with active AS (n = 5) and healthy controls (n = 6). No significant differences were found in the percentage of pho-STAT5^+^ cells or the pho-STAT5 MFI of pho-STAT5^+^ cells between the sorted PB Tregs from the different groups at any time point (Fig. 5b).

Taken together, the above findings indicate that the percentage of phosphorylated STAT5^+^ Tregs did not simultaneously increase with the percentage of STAT5^+^ Tregs. On the contrary, there was a relative decrease in the phosphorylation of STAT5, and STAT5 expression in the sorted PB Tregs from patients with active AS did not reach or maintain equal phosphorylation status in the absence of IL-2 compared with the sorted PB Tregs from healthy controls. These data confirm that abnormal IL-2 signalling exists in PB Tregs in patients with active AS.

### Sorted PB Tregs from patients with active AS had elevated methylation levels in the CpG islands in CNS2

**Figure 6:**
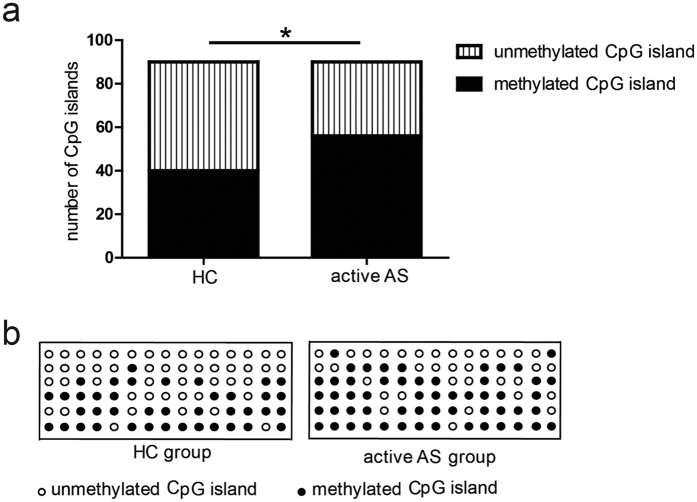
CpG island methylation in Chr X 49260896 in Tregs. (**a**) The number of methylated CpG island clones in 6 HCs and 6 patients with active AS. (**b**) The specific methylation status of the CpG islands in Chr X 49260896 in every sequenced clone from the sorted Tregs from all subjects. Each row represents a HC or an active AS patient, whereas each circle in the row represents a sequenced clone containing target fragments. Open circle: unmethylated clone; filled circle: methylated clone. p < 0.05 indicates significance (Fisher’s exact test); *p < 0.05.

In addition to IL-2 signalling, CNS2 methylation is another main mechanism through which Treg stability is maintained. The methylation levels of the CpG islands on Chromosome X 49260896 in patients with active AS were higher than those in healthy controls (each n = 6) (p = 0.0247) (Fig. 6a). The specific methylation status of the CpG islands is also shown in Fig. 6b, and the sequencing results for the other CpG islands are displayed in Supplementary Table S6. These results suggest that CNS2 methylation is abnormal in active AS and may be underlie the functional defects in PB Tregs that are present in this condition.

## Discussion

In this study, we assessed percentages of PB CD4^+^ T cell subsets in patients with AS and healthy controls and found that active AS is associated with a decrease in FoxP3 MFI in PB Tregs. We then focused on PB Treg function and found that PB Tregs are functionally impaired in active AS. Finally, and importantly, we studied the mechanisms underlying the PB Treg dysfunction found in active AS from two aspects: IL-2 signalling and CNS2 methylation. Our results showed that patients with active AS exhibit abnormal IL-2 signalling and CNS2 hypermethylation in PB Tregs, revealing new pathogenic mechanisms for AS.

Tns differentiate into Th1, Th2, and Th17 cells as well as Tregs in response to TCR stimulation and the presence of certain cytokines^22^. Therefore, we assessed PB Tn activity. Our results demonstrated that Tn proliferation and apoptosis as well as Th17 differentiation from PB Tns did not differ between patients with AS and healthy controls. These results provide new insights into the involvement of T cells in AS pathogenesis. We also found that the percentages of Th1/Th17 cells in patients with AS were not altered, which is consistent with previous reports^23^,^24^. Together with the unchanged frequency of Th2 cells in AS^5^, these results indicate that T-bet and GATA-3, which both have important roles in the differentiation of Th1/Th2 cells^25^, may not involved in AS. Similarly, recent studies have shown that IL-23 is involved in AS through its role in mediating IL-17 production by entheseal resident T cells^26^ and that γ δ T cells may also be involved in AS through IL-17^24^. IL-17 secreted by entheseal resident T cells, γ δ T cells, mast cells, and Th17 cells and may thus play a key role in the development of AS^26^,^27^. We also found that the percentage of classical PB Tregs was unchanged in AS, which is also consistent with the literature^5^,^7^,^8^. The percentage of CD45RA^−^ memory Tregs within the Treg population was also unchanged (see Supplementary Fig. S7).

Of particular interest is the significantly decreased FoxP3 MFI observed in Tregs from patients with active AS, although no difference in *FOXP3* mRNA expression was noted between patients with active AS and healthy controls (each n = 6) (see Supplementary Fig. S4). This decrease in FoxP3 MFI suggests a potential functional impairment of Tregs. Several groups have reported that both continuous and stable FoxP3 expression are needed to maintain the suppressive function of Tregs in genetically engineered mice^18^. Importantly, we found impaired suppressive function in the sorted Tregs from patients with active AS, particularly with regard to limited suppression of Tn proliferation. Although data concerning the functional impairment of PB Tregs in AS are scarce, one report involving only one AS patient indicated such impairment^6^, which provided us with some background information. In our present research, we evaluated 16 patients with active AS, and we are the first to report a statistically significant impairment in PB Treg function in AS. Our results suggest that PB Treg dysfunction is involved in the pathogenesis of AS.

To identify what caused PB Tregs to be defective in patients with AS, we detected IL-2 levels in the supernatants of T cell co-cultures, as IL-2 is important for T cell proliferation and activation^21^,^28^,^29^. We found that patients with active AS had higher IL-2 levels than healthy controls. In our co-culture system, the source of IL-2 was Tns, not Tregs, whereas IL-2 is consumed by both Tns and Tregs^21^,^28^,^29^. Thus, the observed IL-2 enrichment in the co-culture supernatant has three potential causes. The first possibility is that Tns secrete more IL-2 in active AS. However, our data demonstrated that Tns from patients with active AS had the same IL-2 mRNA expression levels as those from healthy controls. The second possibility is that Tns from patients with active AS use less IL-2 than those from unaffected individuals. However, our current data indicated that Tns from patients with active AS showed increased activity after co-culturing, indicating their greater IL-2 usage. The third possibility, and the only plausible possibility based on our results, is that patients with active AS harbour Tregs that are defective in using IL-2, thereby causing them to use less IL-2. As confirmed by this experiment, the reduced utilization of IL-2 can alter Treg function, which represents a new mechanism underlying the pathogenesis of AS.

IL-2 signalling includes both IL-2 usage, or the binding of IL-2 to the IL-2 receptor, and the downstream IL-2 pathway. Stable and sustained FoxP3 expression in mature Tregs depends on the IL-2 signalling pathway^15^. For example, defects in the IL-2 pathway in Tregs contribute to T1D by reducing sustained FoxP3 expression^30^. We therefore measured CD25 (IL-2 receptor a-chain) expression in sorted PB Tregs and found that it did not change in active AS. Second, we measured STAT5, another important downstream molecule in the IL-2 pathway^29^. We found that patients with active AS exhibited higher STAT5 expression in their PB Tregs. Although the percentage of Tregs expressing phosphorylated STAT5^+^ did not increase in these patients, the increased STAT5 expression in Tregs seemed to be compensatory. These results indicate that patients with active AS exhibit inadequate or unstable STAT5 phosphorylation in their PB Tregs. This finding also confirmed that patients with active AS have abnormal IL-2 signalling in PB Tregs, which can affect Treg function.

Another mechanism underlying the maintenance of Treg stability is CNS2 methylation^15^. Demethylation of CpG islands in CNS2 is essential for the maintenance of FoxP3 expression in differentiated mature Tregs^31^. Our data demonstrated that Tregs in patients with active AS have higher CpG methylation levels in CNS2 and that abnormal CNS2 methylation may cause dysfunctions within PB Tregs, promoting the development of AS. To the best of our knowledge, this study is the first to report hypermethylation of CNS2 in active AS.

IL-2 signalling and CNS2 are closely related and can compensate for each other. For instance, CNS2 effectively sustains FoxP3 expression in Tregs when low levels of IL-2 are present, whereas high levels of IL-2 can partially compensate for CNS2 deficiency by activating STAT5 in Tregs^32^. In our studies, defects in IL-2 signalling and increased methylation of CNS2 occurred simultaneously in patients with active AS; hence, the compensation between the two mechanisms was lost, which exacerbate PB Treg dysfunction.

Although we have clearly shown that PB Tregs are functionally impaired in AS, considering the diversity of Tregs^8^,^33^, the pathogenesis of AS may also be affected by other Treg subsets through different mechanisms. One group reported that Tregs from the lamina propria of patients with AS have a suppressive effect on T cells and exhibit increased STAT5 expression^34^, whereas another group demonstrated that the suppressive function of synovial Tregs does not change in AS^7^. Further in-depth studies on the diversity and multiple functions of Tregs may improve understanding of the role of Treg functionality in the pathogenesis of AS. Additionally, because evaluations of different target populations and populations with different ethnic backgrounds may affect results^35^, we evaluated comparable populations of patients with stable AS and patients with active AS. Notably, our study populations showed no significant differences in age, gender or HLAB-27 positivity (Supplementary Table S8).

Collectively, our data indicate that both IL-2 signalling and CNS2 methylation are abnormal in active AS, which causes PB Tregs to become dysfunctional. This finding demonstrates the importance of PB Tregs in the pathogenesis of AS. Given that current routine treatments for AS, including tumour necrosis factor (TNF) blockade, do not actually cure the condition^36^, the identification of more effective treatment approaches is needed. Our findings have created new avenues for investigation into possible cures for AS through the restoration of the normal PB Treg function.

## Patients and Methods

### Patients and healthy controls

Patients were recruited from the Department of Clinical Immunology of Xijing Hospital from December 2012 to July 2015; all patients met the modified New York criteria (1984) for AS^37^. There were 76 patients with AS, and disease activity was evaluated using the Ankylosing Spondylitis Disease Activity Score (ASDAS). An ASDAS ≥ 1.3 was considered active disease, whereas an ASDAS < 1.3 was considered stable disease^38^,^39^,^40^. None of the patients had received immunosuppressive agents (such as methotrexate and sulfasalazine) within three months of the time of sample collection. The study included 42 healthy controls recruited from local volunteers. The mean ± SEM ages of the patients and healthy controls were 28.2 ± 1.04 years and 29.6 ± 1.00 years, respectively, and the male-to-female ratios were 61:15 and 32:10, respectively. The basic characteristics of the patients with AS are shown in Supplementary Table. S8. The Ethics Committee of Xijing Hospital approved this study. Prior to data collection, informed consent was obtained from all patients and healthy controls. All the experiments in this study were performed in accordance with the relevant guidelines and regulations.

### PBMC isolation

PB samples were obtained from all healthy controls and patients with AS. Peripheral blood mononuclear cells (PBMCs) were isolated from the samples via density-gradient centrifugation using Lymphoprep^TM^ (Axis-shield, Norway). The PBMCs were further processed after erythrocytes were lysed with FACS lysing solution (BD Biosciences, American).

### Phenotypic and cell frequency analyses

For analyses of the phenotype and frequency of each cell subset, peridin chlorophyll protein (PerCP)-conjugated anti-CD4, phycoerythrin (PE)-conjugated anti-CD45RO, fluorescein isothiocyanate (FITC)-conjugated anti-interferon (IFN)-γ, allophycocyanin (APC)-conjugated anti-IL-17a, PE-conjugated anti-IL-23R, FITC-conjugated anti-CD4, APC-conjugated anti-CD25, PE-conjugated anti-FoxP3, APC-conjugated anti-CD39 (all from eBioscience, American) and phycoerythrin cyanin-7 (PE-Cy7)-conjugated anti-CD45RA (BD Bioscience) were used. For intracellular IL-17a and IFN-γ staining, cells were stimulated with 50 ng/mL phorbol myristate acetate (PMA) and 1 μg/mL ionomycin (both from Sigma-Aldrich) in the presence of 10 μg/mL GolgiStop (BD Bioscience) for 5 hours at 37 °C in a humidified 5% CO_2_ incubator before staining. The cells were extracellularly stained, then treated with Cytofix/Cytoperm solution and Perm/Wash solution (BD Bioscience). For intranuclear FoxP3 staining, cells were first extracellularly stained and then treated with Fixation/Permeabilization and Permeabilization Buffer (FoxP3 Staining Buffer Set, eBioscience, American). The appropriate isotype controls were used in all the staining procedures, and the stained cells were processed with a BD FACS Calibur and analysed using FlowJo software, version 7.6.1.

### Cell sorting of Tregs and Tns

PBMCs were stained with SYTOX Green (SYTOX® Green Nucleic Acid Stain, Invitrogen), PerCP-conjugated anti-CD4, APC-conjugated anti-CD25 and PE-Cy7-conjugated anti-CD45RA. SYTOX Green^−^ CD4^+^ CD25^-^ CD45RA^+^ naïve T cells (Tns) and CD4^+^ CD25^high^ T cells (sorted Tregs) were sorted via fluorescence-activated cell sorting (FACS) analysis using a BD FACS Aria II (98% purity for each subset).

### Suppression of Tn proliferation by Tregs

Freshly sorted Tns were labelled with 5, 6-carboxyfluorescein succinimidyl ester (CFSE) (CellTrace™ CFSE Cell Proliferation Kit, Invitrogen Ltd) according to the manufacturer’s instructions. The labelled cells (1 × 10^5^) were cultured with or without sorted Tregs (5 × 10^4^) in a 96-well U-bottomed plate in the presence of anti-CD3/CD28 beads (Tns:beads = 1:1). On day 5, supernatant was collected and stored at −80 °C until submitted to an ELISA. The concentrations of IL-10, transforming growth factor (TGF)-β, granzyme B and IL-2 in the supernatants were assessed (all reagents from Biolegend, American).

In addition, on day 5, Tn proliferation was determined based on CFSE fluorescence measurements by flow cytometry.

### Analysis of Tn apoptosis

Sorted Tns were cultured in the presence of anti-CD3/CD28 beads (Dynabeads® Human T-Expander CD3/CD28 bead, Life Technology, American) (cells:beads = 1:1) for 24 or 72 hours. Then, the cells were collected and incubated with Annexin V and propidium iodide (PI) for 15 minutes on ice while protected from light. After being washed with Annexin V Binding Buffer (all from Biolegend, American), the samples were processed on a BD FACS Calibur for further analysis.

### Tn differentiation into Th17 cells

Sorted Tns were activated with anti-CD3/CD28 beads (Dynabeads® Human T-Expander CD3/CD28 bead, Life Technology, American) (cells:beads = 1:1) and cultured under the following Th17 skewing conditions: 20 ng/mL human recombinant IL-6, 1 ng/mL TGF-β1, 10 ng/mL IL-1β, and 50 ng/mL IL-23 (all from HumanZyme, R&D systems, American). The percentages of CD4^+^ IL-17a^+^ Th17 cells and CD4^+^ IFN-γ^+^ Th1 cells were assessed on day 3 and day 7 through extracellular and intracellular staining with the appropriate antibodies, and the results were analysed using FlowJo software version 7.6.1.

### IL-2 gene expression in Tns and *FOXP3, IL-10* AND *TGF-β* gene expression in Tregs

For detection of IL-2 expression in Tns, sorted Tns (1 × 10^5^) were cultured with or without sorted Tregs (5 × 10^4^) in the presence of anti-CD3/CD28 beads (Tns:beads = 1:1) in a 96-well U-bottomed plate. For detection of *FOXP3, IL-10* and *TGF-β* gene expression in Tregs, sorted Tregs (5 × 10^4^) were cultured with sorted Tns (1 × 10^5^) in the presence of anti-CD3/CD28 beads (Tns:beads = 1:1) in a 96-well U-bottomed plate. On day 5, the cells were collected and total RNA was extracted using an RNeasy Mini Kit, after which the RNA was reverse-transcribed into cDNA using a Reverse Transcription Kit (all from Qiagen, Germany). Expression levels of the IL-2 gene in Tns and the *FOXP3, IL-10* and *TGF-β* genes in Tregs were detected by real-time quantitative polymerase chain reaction (qPCR) using a Mx3000 P real-time QPCR system. Four housekeeping genes (*β2 M, RNA18S5, SDHA* and *YWHAZ*) were included. The most appropriate reference genes were *β2 M* and *SDHA*, which were chosen with Normfinder software^41^.

### STAT5 phosphorylation in sorted Tregs

Sorted Tregs were cultured in serum-free medium at 37 °C in a humidified 5% CO_2_ incubator for 2 hours. Then, the cells were cultured for 0, 5, or 30 minutes in the presence of IL-2 (50 U/mL, R&D Systems, American). For detection of STAT5 or phosphorylated-STAT5 (pho-STAT5), the following antibodies were used: an anti-human STAT5 (E289) antibody (ab32043, Abcam, Britain), an anti human pho-STAT5 (Tyr694) (C11C5) rabbit monoclonal antibody (Cell Signaling) and goat anti-rabbit IgG H&L (DyLight® 488, Abcam, Britain). After the sorted Tregs were stimulated with IL-2 for different periods of time, they were fixed with 2% PFA and permeabilized with 90% methanol. Following blockade with goat serum and FcR blocker, the samples were stained with antibodies. Finally, the samples were subjected to analysis with a BD FACS-Calibur.

### Methylation of CNS2 CpG islands in the *FOXP3* gene in sorted Tregs

There are ten CpG loci included in the CNS2 region (from Chromosome X 49260769 to Chromosome X 49260954) of the *FOXP3* gene; the locations of the ten CpG islands on the X chromosome are as follows: 49260842, 49260847, 49260888, 49260896, 49260906, 49260909, 49260915, 49260919, 49260927, and 49260936. DNA was extracted from sorted Tregs and modified with sodium bisulphate, and the CNS2 region was amplified using polymerase chain reaction (PCR) with the forward primer 5′ TTGGGTTAAGTTTGTTGTAGGATAG 3′ and the reverse primer 5′ ACATCTAAACCCTATTATCACAACC 3′. The PCR products were detected by 3% agarose gel electrophoresis, and the CNS2 fragments were gel-extracted. Then, the purification products were cloned into a Puc18-T vector and transformed into competent cells. White colonies were chosen and cultured in medium containing ampicillin at 37 °C overnight. After a colony containing the target gene was identified, the plasmid was extracted and sequenced.

### Statistical analysis

Data are presented as the mean ± SEM, and the significance of differences was calculated using Fisher’s exact test, Student’s paired *t*-test and Student’s unpaired 2-tailed *t*-test. P values less than 0.05 were considered significant. Calculations were performed using GraphPad Prism software, version 6.

## Additional Information

**How to cite this article**: Guo, H. *et al*. Functional defects in CD4^+^ CD25^high^ FoxP3^+^ regulatory cells in ankylosing spondylitis. *Sci. Rep.* **6**, 37559; doi: 10.1038/srep37559 (2016).

**Publisher's note:** Springer Nature remains neutral with regard to jurisdictional claims in published maps and institutional affiliations.

## Supplementary Material

Supplementary Figures and Legends.
