# Supplementary material for: Functional defects in CD4+ CD25high FoxP3+ regulatory cells in ankylosing spondylitis
Source: Sci Rep. 2016 Nov 30;6:37559. doi: 10.1038/srep37559 (PMC5128857; doi:10.1038/srep37559)
Supplement: Supplementary Figures and Legends. [file srep37559-s1.pdf]

Functional defects in $CD4^+$ $CD25^{high}$ $FoxP3^+$ regulatory cells in ankylosing spondylitis

Huifang Guo[1, 2, 3]¶, Ming Zheng[1, 2, 3]¶, Kui Zhang[1, 3]¶, Fengfan Yang[1, 3], Xin Zhang[1, 3], Qing Han[1, 3], Zhi-Nan Chen[2, 3]* & Ping Zhu[1, 3]*

[1]Department of Clinical Immunology, Xijing Hospital, The Fourth Military Medical University, No. 127 West Changle Road, Xi'an, Shaanxi Province, People's Republic of China. [2]Department of Cell Biology, Fourth Military Medical University. [3]National Translational Science Center for Molecular Medicine, Xi'an 710032, China.

Supported by State Key Development Program for Basic Research of China (No. 2015CB553704).

Correspondence and requests for materials should be addressed to Ping Zhu (email: zhuping@fmmu.edu.cn) or Zhi-Nan Chen (email: znchen@fmmu.edu.cn).

SUPPLEMENTARY FIGURES

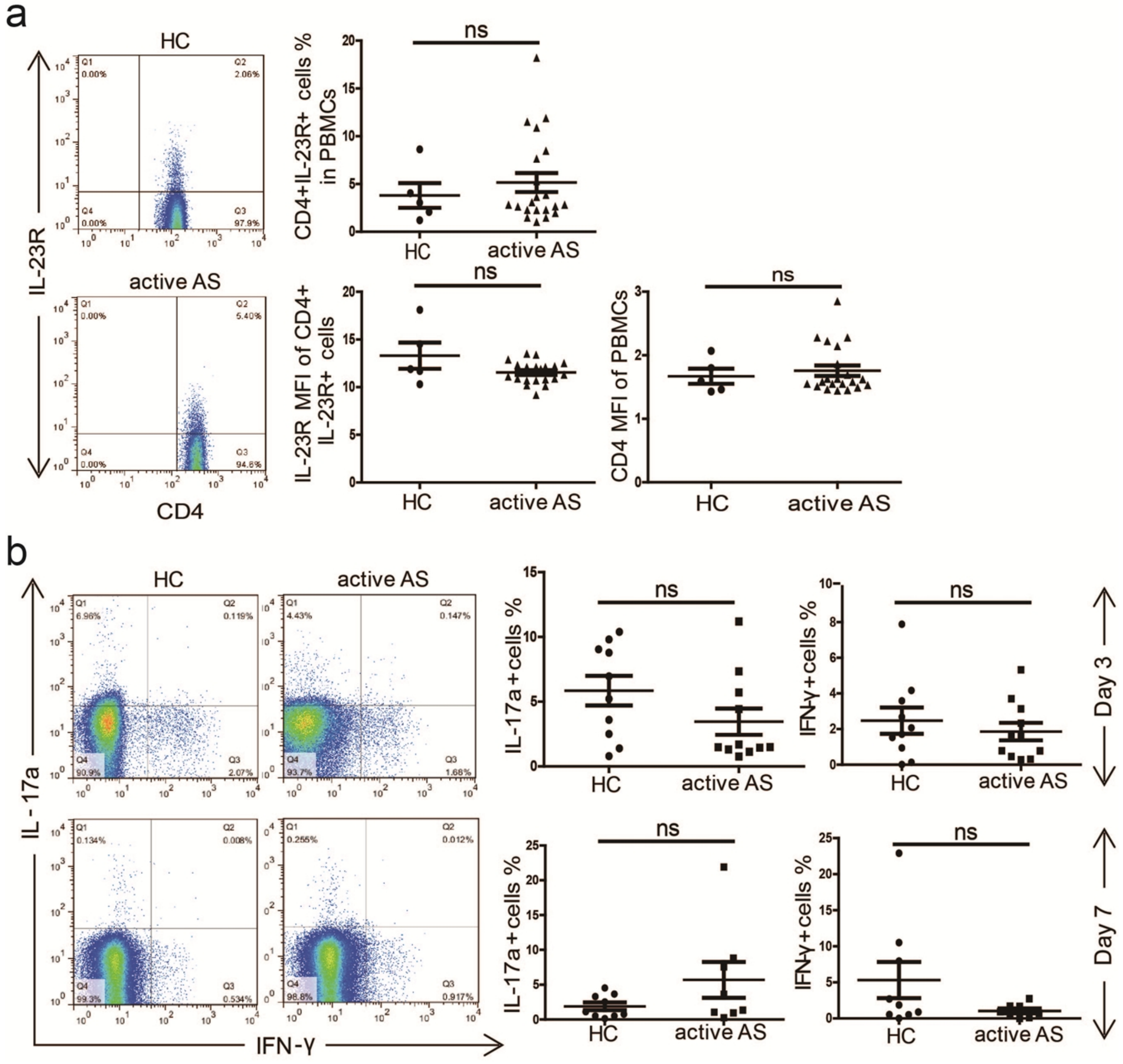

Supplementary Figure S1. Th17 cell differentiation of naïve T cells.

a

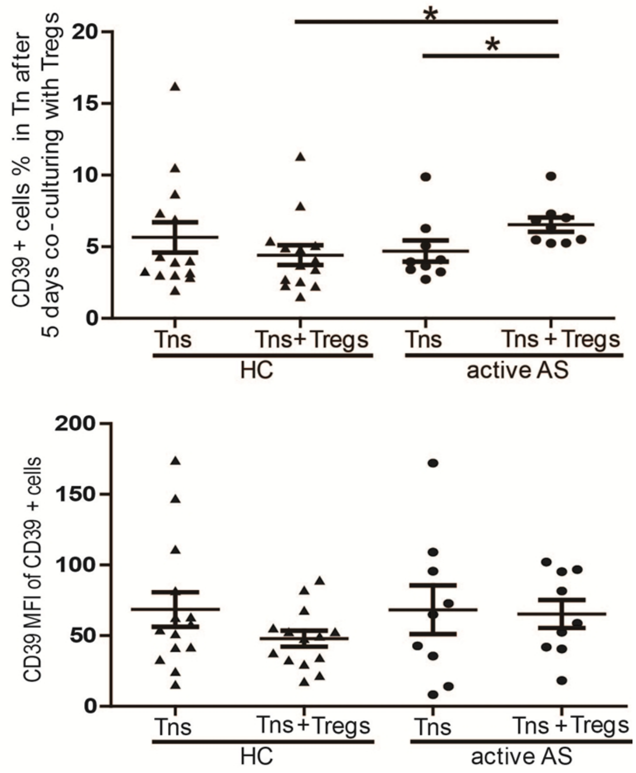

Supplementary Figure S2. CD39 expression by Tns after co-culture with PB Tregs.

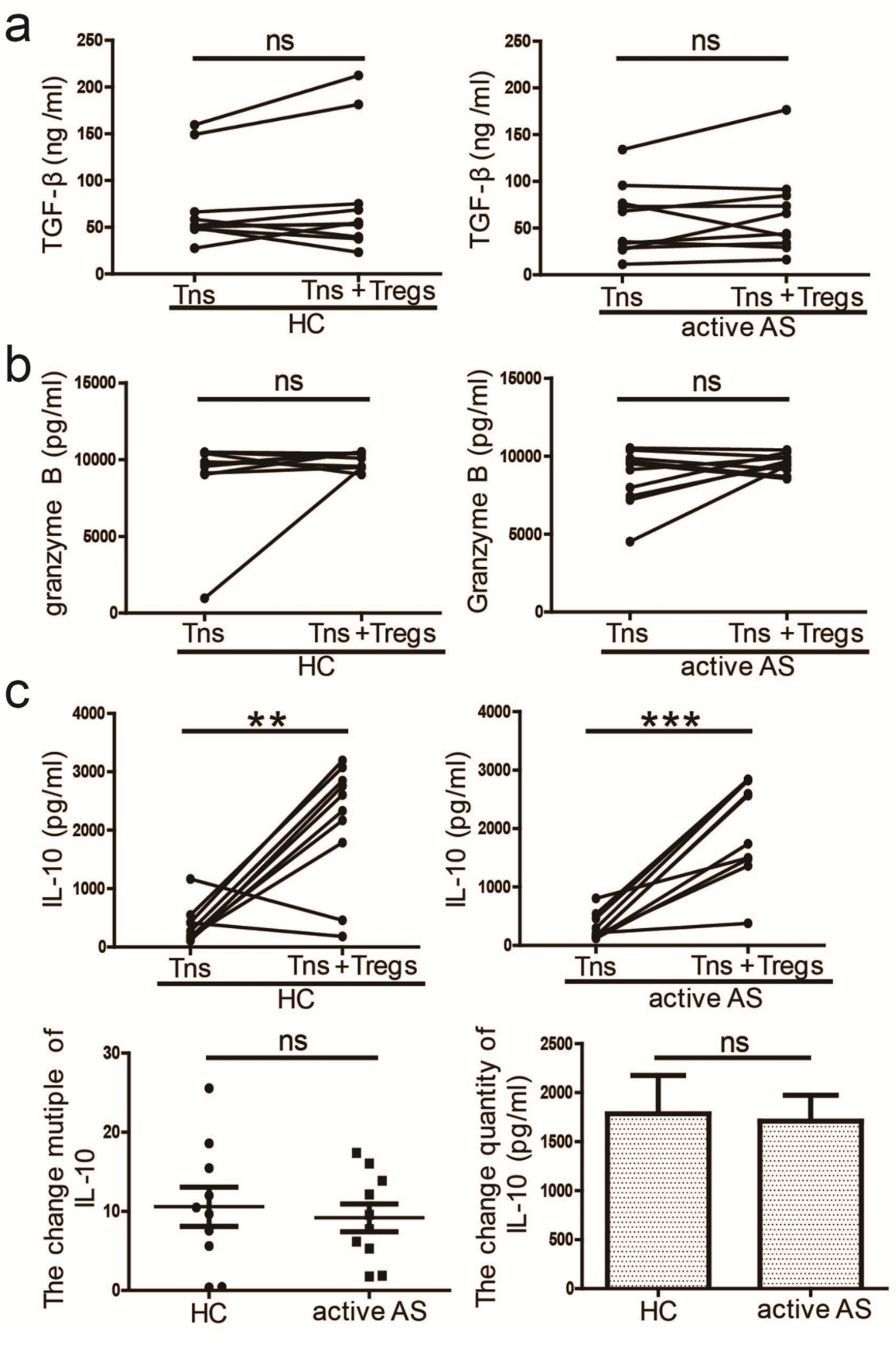

Supplementary Figure S3. The concentrations of cytokines in the cell co-culture supernatants.

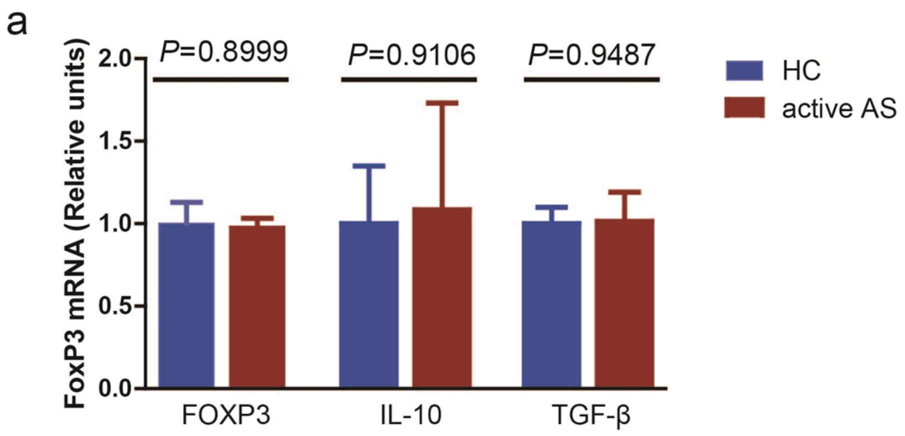

Supplementary Figure S4. The expression of *FOXP3, IL-10* and *TGF-β* in Tregs.

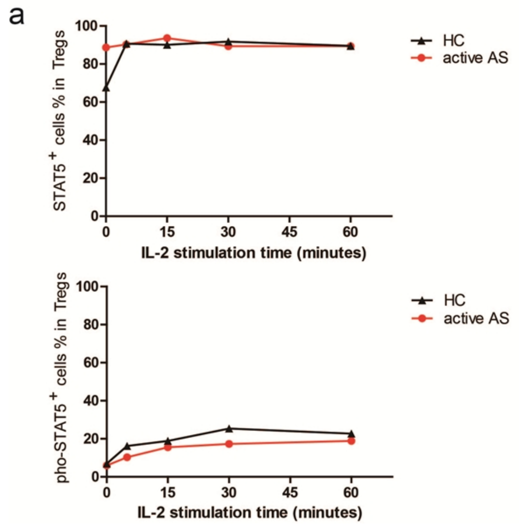

Supplementary Figure S5. The percentages of STAT5+ cells and phosphorylated STAT5+ cells in sorted Tregs following stimulation with IL-2.

Gene/loci of CpG islands	Statistical method	P value	Statistically significant (alpha<0.05)
chrX 49260842	Fisher's exact test Two -sided	P=0.0733	No
chrX 49260847		P=0.5624	No
chrX 49260888		P=0.6541	No
chrX 49260896		P=0.0247	Yes
chrX 49260906		P=0.2264	No
chrX 49260909		P=0.1726	No
chrX 49260915		P=0.1303	No
chrX 49260919		P=0.1303	No
chrX 49260927		P=0.1346	No
chrX 49260936		P=0.1303	No

Supplementary Table S6. Bisulfite sequencing results for the methylated CpG islands.

a

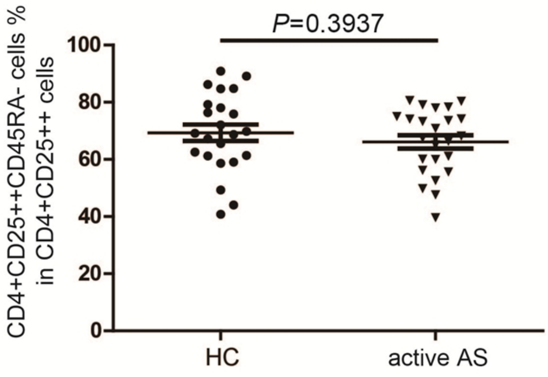

Supplementary Figure S7. The percentage of CD45RA$^-$ memory Tregs in Tregs.

Characteristics	all patients with AS	patients with stable AS (ASDAS < 1.3)	patients with active AS (ASDAS ≥ 1.3)
Patients (n)	76	19	57
Age (years), mean ± SD	28.20 ± 1.04	27.47 ± 1.62	28.44 ± 1.28
Sex (male/female)	64/12	16/3	48/9
HLA-B27 positivity, n (%)	71 (93.42)	18 (94.74)	53 (92.98)
ESR (mm/h), mean ± SD	33.81 ± 3.47	9.31 ± 3.50	41.49 ± 3.85
CRP (mg/dL), mean ± SD	4.92 ± 0.95	0.35 ± 0.14	6.26 ± 1.16
Treatment, n%	0	0	0

Supplementary Table S8. Basic characteristics of the AS patients.

SUPPLEMENTARY FIGURE LEGENDS

Supplementary Figure S1. Th17 cell differentiation of naïve T cells. (**a**) Analyzed was interleukin (IL)-23R expression on $CD4^+$ T cells in peripheral blood mononuclear cells (PBMCs) from healthy controls (HCs) and patients with active ankylosing spondylitis (AS). Percentage of $CD4^+IL\text{-}23R^+$ T cells within PBMC populations, IL-23R mean fluorescence intensity (MFI) of $CD4^+IL\text{-}23R^+$ T cells and CD4 MFI of PBMCs in HCs (n = 5) and patients with active AS (n = 21) are shown. (**b**) Naïve T cells (Tns) from HCs and patients with active AS were cultured under the Th17 cell-skewing conditions (20 ng/mL IL-6, 1 ng/mL transforming growth factor (TGF)-β1, 10 ng/mL IL-1β, and 50 ng/mL IL-23) in the presence of anti-CD3/CD28 beads (Tns:bead = 1:1). The percentage of $CD4^+IL\text{-}17a^+$ Th17 cells in HCs (day 3: n = 10; day 7: n = 9) and patients with active AS (day 3: n = 11; day 7: n = 8) was assessed by flow cytometry. Representative plots of the expression of IL-17a in $CD4^+$ T cells from a HC and a patient with active AS are shown (left). $p < 0.05$ indicates significance (unpaired Student's *t*-test), ns: not significant.

Supplementary Figure S2. CD39 expression by Tns after co-culture with PB Tregs. (**a**) Tns from HCs (n = 14) and patients with active AS (n = 9) were sorted and co-cultured with sorted Tregs (Tns:Tregs = 2:1) in the presence of anti-CD3/CD28 beads (Tns:beads = 1:1). On day 5, the cells were stained with fluorescence-conjugated antibody anti-human CD39 and assessed by flow cytometry. The percentage of $CD39^+$ Tns within Tn populations and the CD39 MFI of $CD39^+$ Tns were assessed. $p < 0.05$ indicates significance (unpaired or paired Student's *t*-test); * = p< 0.05; ns: not significant.

Supplementary Figure S3. The concentrations of cytokines in the cell co-culture supernatants. Tns from HCs and patients with active AS (each n = 10) were treated and cultured as in Figure 3. On day 5, the supernatants were collected. The TGF-β (**a**), granzyme B (**b**) and IL-10 (**c**) in the supernatants of Tns cultured alone or co-cultured with sorted Tregs were assessed. Unpaired Student's *t*-test was used for comparisons between HCs and patients with active AS; otherwise, paired Student's *t*-test was utilized. $p < 0.05$ indicates significance; ** = p< 0.01; *** = p< 0.001; ns: not significant.

Supplementary Figure 4. The expression of *FOXP3, IL-10* and *TGF-β* in Tregs. (**a**) Tregs of HCs and patients with active AS (each n = 6) were sorted and co-cultured with sorted Tns (Tns:Tregs = 2:1) in the presence of anti-CD3/CD28 beads (Tns:beads = 1:1). On day 5, the cells were collected and RNA was extracted and reverse-transcribed into cDNA. The mRNA of *FOXP3*，*IL-10* and *TGF-β* were assessed by real-time quantitative

polymerase chain reaction (qPCR). $p < 0.05$ indicates significance (Unpaired Student's *t*-test).

Supplementary Figure S5. The percentages of STAT5+ cells and phosphorylated STAT5+ cells in sorted Tregs following stimulation with IL-2. (**a**) The sorted Tregs from a HC and a patient with active AS were starved in serum free medium for 2 hours and stimulated with IL-2 for different time (0, 5, 15, 30 and 60 minutes). The percentages of $STAT5^+$ T cells and phosphorylated $STAT5^+$ T cells in sorted Treg populations were assessed.

Supplementary Table S6. Bisulfite sequencing results for the methylated CpG islands. The methylation level of 10 CpG islands was detected, and only the methylation level of Chr X 49260896 CpG was higher in Tregs from patients with AS than that from healthy controls. $p < 0.05$ indicates significance (Fisher's exact test).

Supplementary Figure S7. The percentage of $CD45RA^-$ memory Tregs in Tregs. (**a**) Percentages of $CD4^+CD25^{++}CD45RA^-$ memory Tregs within $CD4^+CD25^{++}$ Treg populations in HCs (n = 23) and patients with active AS (n = 24) are shown. $p < 0.05$ indicates significance (Unpaired Student's *t*-test).

Supplementary Table S8. Basic characteristics of the AS patients. HLA-B27, human leukocyte antigen B27; ESR, erythrocyte sedimentation rate; CRP, C-reactive protein; Treatment, at the time of sample collection the patient had received immunosuppressive agents or biological agents within three months.
